# Factors influencing women’s preference for health facility deliveries in Jharkhand state, India: a cross sectional analysis

**DOI:** 10.1186/s12884-016-0839-6

**Published:** 2016-03-07

**Authors:** Sanghita Bhattacharyya, Aradhana Srivastava, Reetabrata Roy, Bilal I. Avan

**Affiliations:** Public Health Foundation of India, Plot no. 47, Sector 44 Institutional Area, Gurgaon, 122002 Haryana India; Department of Population Health, London School of Hygiene and Tropical Medicine, Keppel Street, London, WC1E 7HT United Kingdom

**Keywords:** Delivery, Childbirth, Maternal health, Quality of healthcare, Cross-sectional survey, India

## Abstract

**Background:**

Expanding institutional deliveries is a policy priority to achieve MDG5. India adopted a policy to encourage facility births through a conditional cash incentive scheme, yet 28 % of deliveries still occur at home. In this context, it is important to understand the care experience of women who have delivered at home, and also at health facilities, analyzing any differences, so that services can be improved to promote facility births. This study aims to understand women’s experience of delivery care during home and facility births, and the factors that influence women’s decisions regarding their next place of delivery.

**Method:**

A community-based cross-sectional survey was undertaken in a district of Jharkhand state in India. Interviews with 500 recently delivered women (210 delivered at facility and 290 delivered at home) included socio-demographic characteristics, experience of their recent delivery, and preference of future delivery site. Data analysis included frequencies, binary and multiple logistic regressions.

**Results:**

There is no major difference in the experience of care between home and facility births, the only difference in care being with regard to pain relief through massage, injection and low cost of delivery for those having home births. 75 % women wanted to deliver their next child at a facility, main reasons being availability of medicine (29.4 %) and perceived health benefits for mother and baby (15 %). Women with higher education (*AOR* = 1.67, 95 % CI = 1.04–3.07), women who were above 25 years (*AOR* = 2.14, 95 % CI = 1.26–3.64), who currently delivered at facility (*AOR* = 5.19, 95 % CI = 2.97–9.08) and had health problem post-delivery (*AOR* = 1.85, 95 % CI = 1.08–3.19) were significant predictors of future facility-based delivery.

**Conclusion:**

The predictors for facility deliveries include, availability of medicines and supplies, potential health benefits for the mother and newborn and the perception of good care from the providers. There is a growing preference for facility delivery particularly among women with higher age group, education, income and those who had antennal checkup. In order to uptake facility births, the quality improvement initiatives should regularly assess and address women’s experiences of care.

**Electronic supplementary material:**

The online version of this article (doi:10.1186/s12884-016-0839-6) contains supplementary material, which is available to authorized users.

## Background

Reducing maternal mortality is a critical global priority. Efforts have resulted in a 47 % decline in global maternal deaths since 1990. Yet, globally an estimated 287,000 maternal deaths continue to occur every year [1]. In addition, 300 million women in the world suffer from long-term or short-term illness brought on by pregnancy or childbirth [2]. One of two indicators used to track progress towards achieving MDG 5 is the proportion of births attended by skilled health personnel [3]. Global policies also aim at shifting the place of delivery from home to hospital as one of the strategies to improve maternal and neonatal survival [4, 5]. This has led to unprecedented increases in facility births in several countries [6, 7].

India is one of the countries to have adopted a conditional cash transfer scheme to encourage facility deliveries, being implemented since 2005. Facility deliveries in India have since expanded from 40.7 % in 2005–06 to 72.9 % in 2009–10 [8]. There is some research evidence associating facility deliveries with a decline in perinatal mortality and still births in India [9]. It has also led to an increase in institutional delivery by at-risk mothers, which has the potential to reduce maternal morbidity and mortality [10]. Expanding health system effectiveness and socio-economic development, along with rising institutional deliveries have brought down maternal mortality ratio (MMR) over the years from 390 in 2000 to 179 in 2012 [2, 11]. However, India still accounts for 19 % of global maternal deaths and is still far from achieving the fifth MDG target of reducing maternal mortality to 100 [2].

One of the reasons for persistently low utilization of facility deliveries in developing countries is women’s preference for at- home deliveries. This preference is attributed to the satisfaction derived from home delivery in comparison with facility delivery based on previous experience, or perceptions surrounding these services [12–14]. Services of traditional birth attendants continue to be widely used as community members believe health services are necessary only when there are complications [15]. On the other hand, there have been reports of unkind treatment by health facility staff [12, 16] poor attitude and absenteeism [14, 17] and overall perceived poor quality of health services, [14, 17–20] as deterrents to seeking facility delivery. A study showed that women with complications would delay or avoid seeking care in a facility if they had a previous experience of disrespectful treatment by staff [14]. Other factors related to the level of satisfaction with, or preference for home over facility deliveries in developing countries include the distance of health facilities, costs, and quicker access to traditional birth attendants [13–17, 21, 22]. Good infrastructure is also an important determinant, especially in facilities. Proper waiting areas, beds, housekeeping services, electricity, water and clean toilets are basic structural requirements which affect women’s satisfaction with services [23–25]. Availability and quality of human resources, medicines, and equipment are also important [26–28]. Prompt care, constant attention, perceived ‘good’ care, sharing of information on her condition with the woman and comforting her are other themes that determine maternal satisfaction [23, 27, 29]. Outcome ultimately determines overall maternal perception. Safety and health of mother and child after delivery significantly influence a positive assessment of care by the mother [24, 28, 30].

Few studies in India have explored women’s preferences for home, public or private health facility for delivery [20]. Some studies have explored determinants of utilization of facility delivery [19, 31, 32]. In India, traditionally medical attention is deemed unnecessary as delivery is believed to be a natural process [12, 13, 19] and an unassisted birth is considered a sign of courage [13]. Sometimes, economic status may influence decisions regarding place of delivery, even more than access, especially when choosing between private and public healthcare. Utilization of private health services is seen as an index of wealth and status [19]. Public health facilities are the main source of affordable facility deliveries for India’s poor, and a good patient experience would be a critical factor for enhancing facility deliveries [17]. There are, however, instances where women expressed a desire for facility delivery but still ended up delivering at home. The most common reason for this has been noted as the rapid progression of labor with no time to reach a facility, thereby leading to home deliveries [16, 17]. Other factors inhibiting facility delivery include resistance by older women relatives, and sometimes women being sent back home from the facility as delivery was not due that day [16]. In spite of the conditional cash incentive scheme introduced in India, there are states like Jharkhand where 60 % of deliveries continue to take place at home [8].

In this context, it is important to understand the experience of care of women who have delivered at home vis-a-vis those who’ve delivered at a health facility, analyzing any differences so that services can be improved, with the goal to promote facility births, particularly in areas where women still prefer to home births. This study aims to understand women’s experiences of delivery care for home and facility births and the factors that influence women’s decisions regarding their next place of delivery.

## Method

### Study site

This study was conducted in one district in the state of Jharkhand in eastern India. Jharkhand is among the least developed states of India. More than half of households in Jharkhand (52 %) fall in the lowest wealth quintile. Age at marriage is low in the state, as about 63 % of women aged 20–24 years got married before the age of 18 [33]. Jharkhand has an MMR of 219, higher than the national average of 178 [34]. In terms of maternal health indicators, the state shows a dismal picture as only 11 % of women in the state who delivered during the 12 months preceding the survey received full antenatal care and only 40 % deliveries in the state are conducted in health facilities, which is also below the national average of 73 % [8].

### Study design and survey instrument

A cross-sectional community-based study design was selected. The data was collected in a six-week period in April and May 2012. The women were selected according to the following criteria: (i) women who had normal deliveries and (ii) women who delivered in a public health facility or at home with the support of a Traditional Birth Attendant (TBA). Women who had complicated deliveries, whether assisted or C-section, were excluded to obtain a homogeneous sample as women with complicated deliveries would have had a different experience of care. In order to reduce recall errors as far as possible, women who had delivered within 42 days before data collection were interviewed retrospectively at the community.

A structured questionnaire with close-ended questions was developed to capture the experience of care and satisfaction with maternal health services (Addition file 1). It was based on the determinants of maternal care identified from a literature review and qualitative study conducted before the survey [35, 36]. The process helped in identifying determinants of maternal care across infrastructure, the process of care and outcomes [items listed in Table 2]. Among structural elements, good physical environment, cleanliness, availability of adequate human resources, medicines, and supplies emerged as key determinants. Determinants of the process of care included respectful behavior, privacy, promptness, cognitive care, perceived provider competency, and emotional support. Outcome related determinants included health status of the mother and newborn. Access and cost were also included. Covariates captured contextual factors like a woman’s socio-economic status (age, religion, caste, family type, and household income), reproductive history (parity, place of last delivery) and her experience of antenatal and postnatal care. Evidence from the review showed that maternal characteristics like age and parity affected women’s perceived experience of care [37–39]. Ethnicity and literacy were also significant predictors of maternal satisfaction in developing countries [27, 39, 40].

Along with capturing women’s experiences with care, they were further asked whether they were willing to deliver at the same place (facility or home) in the future (for their next delivery). The questionnaire was translated into the local language (Hindi and Bengali) and back-translated to check the linguistic accuracy. The translated questionnaire was reviewed by a panel of national and regional experts in maternal health to validate the content and contextual relevance. The questionnaire was also pretested in a similar population setting to confirm the content, ease of administration and comprehension by local women.

### Sampling and sample size

A list of all women who had given birth in the study district between 15th December 2011 and 31st March 2012 was generated, which included 2377 women. The list comprising the place of birth (home or facility birth), date of delivery, respondent’s address was obtained from the immunization tracking register maintained by the Auxiliary Nurse Midwife (ANM). This register forms part of the routine report for the Health Management Information System (HMIS) under the National Rural Health Mission (NRHM), Government of India. To cross check the completeness of the register, a random check of 5 % of the data was done in the community by contacting the ASHA (community-level health worker) who collects the information for the immunization register. The sample size calculated for the study was 535 with 80 % power and 95 % confidence interval. The sample was further stratified by place of delivery, based on the proportion of women who delivered at a health facility [8]. Women in the final sample were selected randomly with 40 % of women having delivered at a health facility and the rest having home births. The randomization was conducted using Microsoft Excel software. Selected women were approached for interviews at their homes. Community health workers of the respective villages helped trace the selected women. There were 20 refusals and 15 women could not be traced during the survey so a final sample of 500 women (210 women who had delivered at a health facility and 290 who had delivered at home) gave their informed consent to participate in the study.

### Data collection

The instrument was translated into Hindi and Bengali, the local languages spoken in the study area and was pretested among a similar population. An individual who had good knowledge of the local language translated the English version into the local language for better comprehension. It was also back-translated to check for its original meaning. Ten women data collectors with a social science background and three supervisors were trained for 5 days on the study instrument and data collection procedure. At the time of the interview, an informed consent form was read and explained to the respondents. As more than half of the respondents in the study area were illiterate and the literates had limited writing skills with just four mean years of schooling, verbal consent was taken from the participants. Response to informed consent was recorded in the participant information sheet appended to the questionnaire. Confidentiality was assured and maintained during the data analysis.

The study received ethical approval from Public Health Foundation of India’s Institutional Ethics committee (TRC-IEC 112/11).

### Data analysis

The software Epi Info Version 7 was used for data entry. Double data entry was conducted. The data was analyzed using PASW Statistics 17 (formerly known as SPSS). Women’s experience of care during delivery was explored by using cross-tabulation and chi-square tests. To understand women’s preference for future delivery place multiple logistic regression analysis was done taking into factors like ethnicity, socio-economic status and reproductive history of women.

## Results

Table 1 presents the socio-economic, demographic and pregnancy-related characteristics of the respondents. The mean age of women interviewed was 24 years, with 52 % belonging to the age-group of 19–24 years (Table 1). They were predominantly Hindu, with Muslims and others constituting only a fifth of the total respondents. Socially vulnerable caste and tribe groups in India are constitutionally categorized as Scheduled Castes and Scheduled Tribes respectively while ‘general castes’ refer to the non-vulnerable groups. About 80 % of the women belonged to Scheduled Castes and Tribes (74 %) and Other Backward Castes (6 %). Literacy levels were low with 54 % women without any formal education. Most women lived in joint families (69 %) and belonged to households falling in the two lowest income quintiles (86 %). Among pregnancy characteristics, the parity was 2.4 and the mean number of living children per woman was 2.2. The sex of the index child was a boy for 49 % women and girl for 51 % women. The study found significant differences between women with home and facility births for age, caste, religion, education level, type of household, parity, and number of living children.

**Table 1:** Characteristics of the sampled women, India

Characteristics		Respondent n, (%)		
		Facility births (*N* = 210)	Home births (*N* = 290)	Total (*N* = 500)
Socio-demographic and economic profile				
Age (years)***	Mean (SD)	23.01 (3.7)	25.13 (4.4)	24.2 (4.3)
Education***	Illiterate	81 (38.6)	186 (64.1)	267 (53.4)
	Literate	129 (61.4)	104 (35.9)	233 (46.6)
	Mean Years of Schooling (SD)	4.52 (4.4)	2.17 (3.4)	3.15 (4.0)
Religion***	Hindu	146 (69.5)	250 (86.2)	396 (79.2)
	Muslim	53 (25.2)	29 (10)	82 (16.4)
	Traditional	11 (5.2)	11 (3.8)	22 (4.4)
Caste**	General	28 (13.3)	74 (25.5)	102 (20.4)
	Other Backward Caste	11 (5.2)	17 (5.9)	28 (5.6)
	Scheduled Caste/Scheduled Tribe	171 (81.5)	199 (68.6)	370 (74)
Type of Household***	Nuclear	41 (19.5)	112 (38.6)	153 (30.6)
	Joint	169 (80.5)	178 (61.4)	347 (69.4)
Household Income (Quintiles)	Q1-Lowest	18 (8.6)	37 (12.8)	55 (11.0)
	Q2	153 (72.9)	216 (74.5)	369 (73.8)
	Q3	31 (14.8)	31 (10.7)	62 (12.4)
	Q4	7 (3.3)	6 (2.1)	13 (2.6)
	Q5 -Highest	1 (0.5)	0	1 (0.2)
Reproductive History				
Parity, Mean (SD)***		2.03 (1.3)	2.70 (1.6)	2.42 (1.5)
No. of living children, Mean (SD)***		1.81 (1.2)	2.49 (1.4)	2.20 (1.3)
Sex of last baby	Male	102 (48.6)	141 (48.6)	243 (48.6)
	Female	108 (51.4)	149 (51.4)	257 (51.4)

*denotes *p* < 0.05, **denotes *p* < 0.01 ***denotes *p* < 0.001

### Experience with care during childbirth

Table 2 presents findings related to women’s experience of care during delivery. Among structural aspects of care, most women found the delivery table to be clean (90 %). Only a quarter (24.5 %) of women received pain relief medication in home deliveries. A higher proportion of women delivering at home had a good experience with pain management through massage (58 %) as compared to facility deliveries (19 %). The association for both these variables was statistically significant (*p* < .01). Most women who had delivered at the health facility had a good experience with the time taken by providers to attend after the onset of labor (82 %). In the case of home deliveries, a slightly lower proportion of women had good experience with the time taken by the provider to attend (79 %), which in their case was a traditional birth attendant (*p* = 0.239). Among other process indicators, more than 90 % women had good experience with privacy and information shared by birth attendant about the progression of labor and delivery procedures, with little difference between facility and home births (*p* = 0.99 and 0.59 respectively). The difference in outcome for both mother and newborn was also not significant between home and facility deliveries. But the cost of care for facility births is almost three times more than that of home births.

**Table 2:** Experience with care during childbirth for facility and home deliveries among women in India

Dimensions of Care		Experience with care during delivery (%)		
		Facility births (*N* = 210)	Home births (*N* = 290)	*P* Value
Structure (Accessibility, availability of medicine, supplies and hygiene)	Reached facility within 30 min	35.2	NA	NA
	Pain management through injection	54.8	24.5	0.000
	Pain management through massage	19.0	58.3	0.000
	Clean delivery table	90.4	NA	NA
Process of Care (Promptness, Cognitive, privacy, respect by staff who conducted the delivery)	Less than 30 min taken by provider to attend after onset of labor	82.9	78.6	0.239
	Not left alone in delivery room	94.3	93.1	0.594
	Received information about progression of labour	92.4	92.4	0.989
	Received information about delivery procedures	94.3	93.1	0.594
	Delivery in a secluded place and absence of male members	99.1	97.6	0.225
	No abuse during delivery	87.6	92.8	0.052
Outcome (morbidity and mortality)	No health problem of women post delivery	65.3	67.2	0.640
	No health problem of new born post delivery	88.5	86.5	0.502
Other (cost)	Average expenditure in INR	2138	797	NA
		(1375–2901)	(689–904)	

*NA* not applicable

### Willingness to deliver at health facility in the future

Most women preferred having their next delivery at a health facility. Among women who delivered at institutions, an overwhelming 90 % said they would deliver again at a facility in future. Even among women who delivered at home, almost two-thirds (63 %) wanted to deliver at a facility in future (*p* < .01). (Table 3).

**Table 3:** Preference regarding place of future delivery among women in India

Place of current Delivery	Willingness to deliver at health facility in the future				*P* value
	Yes		No		
	N	(%)	N	(%)	
Health facility	189	(90.0 %)	21	(10.0 %)	.000
Home	183	(63.1 %)	107	(36.9 %)	.000
Total	372	(74.4 %)	128	(25.6 %)	.000

#### Factors determining health facility delivery in future

All women were asked whether they wanted to deliver their next child in a public health facility. We conducted a multiple logistic regression analysis with future facility delivery as the dependent variable and selected indicators for socio-economic status and reproductive history of women as independent variables (Table 4). Place of last delivery, mother’s age, education and occurrence of maternal health problem post-delivery emerged as significant predictors of facility delivery in future. The odds of future delivery at a public health facility is 5.19 times higher for women who had institutional delivery (compared to those who had home delivery), 1.67 times more for literates (compared to illiterate women), 2.14 times higher for women in the age group 25 and above (compared to those below 25 years), and 1.85 times more for those who developed a health problem post-delivery (compared to women without any health problem). The unadjusted odds of monthly household income (above INR 5000 versus up to INR 5000), type of family (joint versus nuclear), and antenatal checkup (at least one checkup versus no checkup) appeared significant, however, these were not reflected in the adjusted odds ratio calculation.

**Table 4:** Odds ratio of factors for future delivery at public health facilities among women in Jharkhand, India (*N* = 500)

Exposure variables	Respondent n (%)	Unadjusted OR (95 % CI)	*p* value	Adjusted^a^ OR (95 % CI)	*p* value
Age					
Less than 25	271 (54.2)	1		1	
25 years and above	229 (45.8)	1.02 (0.68–1.53)	0.890	2.14 (1.26–3.64)	0.005
Caste					
General Caste	102 (20.4)	1		1	
Backward caste	398 (79.6)	0.81 (0.48–1.36)	0.420	0.62 (0.35–1.09)	0.091
Education					
Illiterate	267 (53.4)	1		1	
Literate	233 (46.6)	2.26 (1.48–3.45)	0.000	1.67 (1.04–2.70)	0.032
Monthly household income (INR)					
Up to 5000	424 (84.8)	1		1	
Above 5000	76 (15.2)	2.00 (1.04–3.85)	0.030	1.51 (0.74–3.07)	0.250
Family type					
Nuclear	153 (30.6)	1		1	
Joint	347 (69.4)	1.90 (1.25–2.89)	0.002	1.50 (0.94–2.41)	0.082
Parity					
Primi	162 (32.4)	1		1	
Multi	338 (67.6)	0.68 (0.44–1.07)	0.10	0.83 (0.47–1.48)	0.543
Place of delivery of last child					
Home	290 (58.0)	1		1	
Facility	210 (42.0)	5.26 (3.16–8.76)	0.000	5.19 (2.97–9.08)	0.000
Antenatal check up by skilled provider					
Did not receive antenatal check up	28 (5.6)	1		1	
Received antenatal check up (at least one)	472 (94.4)	4.28 (1.96–9.33)	0.000	2.26 (0.96–5.28)	0.061
Mother’s health post delivery					
No health Problem	346 (69.2)	1		1	
Health problem	154 (30.8)	1.82 (1.14–2.92)	0.010	1.85 (1.08–3.19)	0.023
Newborn health post delivery					
No health Problem	441 (88.2)	1		1	
Health problem	59 (11.8)	1.39 (0.71–2.72)	0.322	1.02 (0.47–2.17)	0.951

^a^Adjusted for other variables given in the table

### Reasons for willingness to deliver at health facility in future

The majority of the women wanted to deliver at a facility. Table 5 presents the reasons for their willingness to deliver their next child at the facility. Women could select the primary reason for preferring to deliver their next child at a facility from eight options, derived from the dimensions of the structure, process and outcome of care. Availability of supplies and medicines was one of the major reasons why most women who had delivered in a facility (39 %) and at home (40 %) were willing to deliver at a health facility in future (*p* = 0.025) (Table 5). Good care and support from providers was also an important reason for women’s willingness to deliver at a facility in future (16 and 15 % for facility and home deliveries respectively) (*p* = 0.111). The perception of health benefits for mother and child was also an important reason (25 and 15 % for facility and home deliveries respectively) (*p* < .001). Cost was also an important consideration while choosing the place of delivery and 12 and 22 % women who had delivered in facility and home respectively were willing to deliver at a facility in future as they perceived the cost as reasonable (0.468).

**Table 5:** Reasons for willingness to deliver at health facility for next delivery among women in India

Themes of care	Primary reason	Place of previous delivery		*P* value
		Institution (*n* = 189)	Home (*n* = 183)	
		Frequency (%)	Frequency (%)	
Structure	Accessibility of the facility	8 (4.2 %)	6 (3.3 %)	0.244
	Medicine and Supplies	73 (38.6 %)	74 (40.4 %)	0.025
	Infrastructure	3 (1.6 %)	1 (0.5 %)	0.179
	Skilled staff	3 (1.6 %)	5 (2.7 %)	0.795
Process of care	Good care (cognitive, emotional support, inter-personal care) from providers	30 (15.9 %)	28 (15.3 %)	0.111
	Personal comfort	2 (1.1 %)	2 (1.1 %)	0.745
Outcome	Health benefits for women and newborn	47 (24.9 %)	27 (14.8 %)	0.000
Others	Reasonable cost	23 (12.2 %)	40 (21.8 %)	0.468

## Discussion

The study findings show that there is no major difference in the experience of care between home and facility births, the only difference in care being with regard to pain relief through massage/medicine and the low cost of delivery for those having home births. Findings show a growing preference for facility delivery among women in the study area. The major reasons for this preference were the availability of good supplies i.e. medicine and the perceived health benefits for mother and baby.

In South and Southeast Asia, even recently, more than 70 % of all births in the lowest two wealth quintiles occurred at home [13]. Women consider the Traditional Birth Attendant (TBA) as a culturally acceptable and competent health worker [41]. Residing in the same community, she offers respectful, prompt care and personalized attention to the woman during delivery [36]. Poor accessibility of the institution and factors like lower maternal education, being a rural resident and multi-parity increase the likelihood of home delivery [13, 14, 17, 21, 42]. On the other hand, evidence from developed countries shows that convenience, privacy, and respect are also very important determinants of a woman’s preference for home deliveries [12, 16, 41]. However, the findings of this study show that there is no major difference in the experience of care between facility and home births, in terms of promptness and respectful care. For process of care indicators like time taken by provider to attend after onset of labor, women not being left alone in delivery room, sharing of information about progression of labor, delivery conducted in a secluded place and absence of male members, the experience of care was same for women who have delivered at home and at the facility.

This study’s findings show that a significant proportion of women, who delivered either at a facility (74.4 %) or at home (63 %), were inclined to deliver their next child at the health facility. One of the main reasons for their preference is that they perceive better availability of medicines and supplies, and improved health outcomes of both mother and newborn at facilities. There are other studies from India which have shown that safety of mother and child was the prime concern for households opting for institutional delivery, along with monetary incentives under the conditional cash transfer scheme, and motivation by community health workers [43]. Utilization of public facility is also related to socio-cultural factors like religion, caste, age, education and economic condition [43, 44].

Findings for unadjusted odds ratios in this study show that women with higher education level, belonging to higher age group, who have previously had facility delivery, and those with health problems post-delivery were more likely to give birth at a health facility in the future. Unadjusted figures show those who have received antenatal checkup, belonging to higher income level, and living in joint family, are also more likely to deliver their next child at a health facility. Other studies in similar settings have also shown that the number of ANC visits is an important factor leading towards delivery at an institution, as during ANC visits it is most likely that the women receive counseling from providers about the importance of safe delivery and are encouraged to deliver at a health facility [45–47]. Controlling for other variables, this study found that the mother’s education, age, and current place of delivery are the factors that strongly predict their decision on the place of future delivery. This finding is consistent with earlier studies, which have shown educational status of mothers to be one of the most significant associated factors for utilization of institutional delivery [41–43, 46, 48–52]. Our study also showed that mothers whose last delivery was at a facility and who had a good experience of care were more likely to go again for a facility delivery. This highlights the need for sustaining the quality of technical as well as interpersonal care in institutions [43]. Particularly, women’s perspective needs to be taken into account in facility quality improvement initiatives to make services more responsive to their needs. Women’s preference towards facility delivery is a significant positive aspect and measures need to be taken to address their preferences so that there is improved utilization of services.

### Limitations

The multivariate analysis includes selected socio-economic and demographic correlates, while other programmatic, institutional; accessibility and cultural factors could also affect delivery place preference. [49]. Although special attention was paid to retrieve the lists of recently delivered women from health workers, there is a possibility some women might have been missed out, especially those who delivered at home. The responses could suffer from recall bias. The study did not consider women who had a complication during pregnancy and pertains to only normal deliveries. This was done to have a homogeneous sample as women in the above-mentioned category would have had a different experience of care. The study did not explore all dimensions of respectful care and limited its inquiry to the experience of any abuse in the form of shouting, using abusive language, delivery in a secluded place and not being left alone in the delivery room.

## Conclusion

This study is a community-based survey to assess the predictors of facility deliveries in rural eastern India, which is relevant to other developing countries with a high proportion of home births. In spite of a government-led cash incentive program to promote institutional delivery, a significant number of deliveries are happening at home in India [8]. The reasons for women’s willingness to deliver at a facility include the availability of medicines and supplies, potential health benefits for the mother and newborn and the perception of good care from the providers. This is aligned with women’s experience of care where they have stated better management of pain during labor at the facility than at home. Women who are more educated with higher income levels, belong to a higher age group and have had an antenatal check-up are more likely to deliver at a health facility. Improving the quality of public maternal health services, particularly the factors that determine women’s decision, if assessed regularly and incorporated as part of quality improvement programs at the facility can result in increased maternal satisfaction, thus improving the demand for facility births.

## Additional file

Additional file 1: Questionnaire for quantitative survey. (PDF 397 kb)
