# Supplementary material for: Factors influencing women’s preference for health facility deliveries in Jharkhand state, India: a cross sectional analysis
Source: BMC Pregnancy Childbirth. 2016 Mar 7;16:50. doi: 10.1186/s12884-016-0839-6 (PMC4782569; doi:10.1186/s12884-016-0839-6)
Supplement: Additional file 1: — Questionnaire for quantitative survey. (PDF 397 kb) [file 12884_2016_839_MOESM1_ESM.pdf]

Women's Perception of Quality and Satisfaction with Maternal Health Services
Questionnaire for Quantitative Survey

Introduction story: Namaste. We are from an organization- PHFI in Delhi. Our organization studies health related problems faced by people in India. We are studying types of services that women like you have received during pregnancy and delivery given by Sahiya, nurse, dai. Women in your village and the community around you would also be using these facilities for their deliveries or you may also use the facilities for your next delivery. The information provided by you on your experience will help in finding out how your experience and that of other women/ your friends/ other women in your family in the community can be made better. This is help you and other women in your community to have a better health and healthy baby.

ID Number: __________
Date of Interview: ______________
Name of interviewer: _____________

Name of respondent: _________________
Address of respondent: ________________

GENERAL INFORMATION				
**Sl.No**	**Question**	**Coding Categories**	**Skip to**	**Answers**
1.	What is your age?			
2.	What is your Religion?	01 Hindu<br>02 Muslim<br>03 Christian<br>04 Traditional<br>05 Other		
3.	What is your Caste?	01 General<br>02 SC<br>03 ST<br>04 OBC		
4.	What is your education level?	01 Illiterate<br>02 can read and write<br>03 Attended school		
5.	Total years of schooling<br>Exact years			
6.	Monthly income	01 Less than 1000<br>02 1000-5000<br>03 5001-10000<br>04 10000-50000<br>05 50000+		
7.	Type of Family<br>Definitions :<br>Nuclear: Living with only Husband and children<br>Extended: Living with in- laws/ parents and other family members	01 Nuclear<br>02 Extended		
8.	Family/household size<br>Exact Number			
9.	No. of pregnancies (including live birth, miscarriage and still birth)			
10	Number of living children			
11	Outcome of last pregnancy	01 Live birth<br>02 Still birth		
12	i. Current status of newborn : Alive<br><br>ii Sex of the newborn	01 Yes<br>02 No<br><br>01 Male<br>02 Female		
13	Date of last delivery (mm/yy)			
	**For your last pregnancy**			
14	Did you receive any Antenatal care?<br>**User**: Those received service from health system provider (doctor, nurse, ANM, ASHA)<br>**Non user** : received service from Dai, family members	01 Yes<br>02 No	Based on user and non user fill ANC section	
15	Where did you deliver?	01 Facility<br>02 Home		

16	Did you receive postnatal care?<br>**User**: Those received service from health system provider (doctor, nurse, ANM, ASHA)<br>**Non user** : received service from Dai, family members	01 Yes<br>02 No	Based on user and non user fill PNC section	
17	Interview	01 Agreed<br>02 Refused		
18	Reason for refusal			

ANTENATAL CARE
User: Those received service from health system provider (doctor, nurse, ANM, ASHA)
Non user : received service from Dai, family members
[ANC Users only]

#	Question	Coding Categories	Skip to	Answers
	**Background Information**			
19	Who did you receive Antenatal care from?<br>Note only the primary care provider and prompt the option	01 Doctor- Public Health facility<br>02 Doctor- Private<br>03 Nurse<br>04 ANM<br>05 SAHIYA<br>06 Dai<br>07 Other (Specify)		
20	When was your first ANC check-up at health facility?	01 1-3 months of pregnancy<br>02 4-6 months of pregnancy<br>03 7-9 months of pregnancy		
21	How many times did you visit a health facility for ANC check-up?<br>Exact no.			
22	How many times did a health professional/SAHIYA visit you during the antenatal period<br>Exact no.			
	**Accessibility of health facility** *[ANC Users Only]*			
23	What type/level of facility was this?<br>Note only the facility she visited most for ANC	01 Anganwadi centre<br>02 Health- sub-centre<br>03 PHC<br>04 CHC / FRU<br>05 District hospital<br>06 Private facility		
24	How did you usually get to this facility?	01 On foot<br>02 Vehicle (paid)<br>03 Vehicle (free)		
25	How long did it usually take you to reach this facility?	01 More than 2hrs<br>02 1hr to 2hrs<br>03 30mins to1hr<br>04 Less than 30mins		
	**Structure**<br>*[ANC Users Only]*			
26	Did the health facility appear well cleaned?	01 Yes<br>02 No		
	**Supplies** *[ANC users Only]*			
27	How much did you have to pay for:			
	i. Transport			
	ii. Drugs and Injections			
	iii. Tests			
	iv. Consultation			
	vi. Other costs (specify)			
28	Were you given any cash benefit for registering for your ANC?	01 Yes<br>02 No	-29<br>-30	
29	How much did you receive?<br>Exact amount			

	**Human Resources** *[ANC users Only]*			
30	Who usually attended you for facility ANC?<br>Note only the primary care provider and prompt the option	01 ANM<br>02 Doctor<br>03 Nurse<br>04 Anganwadi worker<br>05 Other (specify)		
	**Emotional Support** *[ANC Users Only]*			
31	Who usually accompanied you to the facility?<br>Prompt the option	01 Husband<br>02 Other family member(s)<br>03 Friend(s)/Neighbour(s)<br>04 SAHIYA<br>05 Went Alone<br>06 Other (Specify)		
32	Were you made to feel comfortable by all the staff at the facility?	01 Yes<br>02 No		
33	What was the gender of the staff who usually attended to you during your visits?<br>Note the gender of primary care provider	01 Male<br>02 Female		
	**Cognitive Support** *[ANC Users Only]*			
34	Did the health professional (doctor, nurse or midwife) or SAHIYA ever discuss with you on **Birth preparedness**:			
	i. Maintaining frequency of your ANC visits (at least four times during pregnancy)	01 Yes<br>02 No		
	ii. Identifying a skilled provider and making arrangements for delivery of your child	01 Yes<br>02 No		
	iii. Making a plan/arrangements for transportation to a facility during labour	01 Yes<br>02 No		
	v. Recognising danger signs of serious health problems during pregnancy, childbirth or soon after	01 Yes<br>02 No		
	vi. Knowing where to go and what community resources such as emergency transport, funds, communications are available in case of emergencies	01 Yes<br>02 No		
	vii. Identifying and making arrangements for a blood donor	01 Yes<br>02 No		
	Vii Information about benefit of JSY and associated monetary benefit	01 Yes<br>02 No		
35	Do you think the entire information is/was adequate to your needs?	01 Yes<br>02 No		
36	Did the health professional (doctor, nurse or midwife) or SAHIYA seem willing to answer any questions you may have had?	01 Yes<br>02 No		
	**Promptness** *[ANC Users Only]*			
37	How long did you usually have to wait before seeing your provider?	01 0-15 minutes<br>02 15-30 minutes<br>03 30mins to 1hr<br>04 More than 1hr		
38	Were you satisfied with the time you usually had to wait to be seen?	01 Yes<br>02 No		
	**Confidentiality and Privacy** *[ANC Users Only]*			
39	Were you satisfied with the	01 Yes		

	amount of privacy and confidentiality you were offered during your visits?	02 No		
**Inter-personal aspect of care** *[ANC Users Only]*				
40	Did any staff ever speak to you in a way that upset you? (e.g. shouting, using abusive language	01 Yes<br>02 No		
41	Did you feel comfortable/free to discuss all your concerns with your provider?	01 Yes<br>02 No	If no, ask 42	
42	If no, what do you think would have made you more comfortable with your provider	01 female gender<br>02 male gender (refer to 33)<br>03 someone I am familiar with<br>04 someone more friendly<br>05 other __________		
**Continuum of care/Interaction with SAHIYA** *[ANC Users only]*				
43	Did your SAHIYA ever encourage you to attend monthly clinics, (VHND, ANC etc)	01 Yes<br>02 No		
44	Did your SAHIYA ever help you in accessing services (transport, getting drugs and injections etc) during your ANC?	01 Yes<br>02 No		
45	Did your SAHIYA ever discuss with you and your family on:			
	i. Nutrition	01 Yes<br>02 No		
	ii. Rest	01 Yes<br>02 No		
	iii. Protection from malaria	01 Yes<br>02 No		
	iv. familiarizing yourself with facility	01 Yes<br>02 No		
46	Did you feel adequately prepared for your delivery after your entire ANC	01 Yes<br>02 No		
**Outcome** *[ANC Users 47,48 and Non-Users, 47, 49]*				
47	How would you rate your health after the antenatal period?	01 Very good<br>02 Good<br>03 Average<br>04 Poor<br>05 Very poor		
48	What made you decide to attend ANC for your pregnancy?<br><br>First ask her un-promptly and based on her response note the primary and secondary reason.<br>If she is unable to answer, prompt the response.	**01** Perceived benefits of ANC on pregnancy outcome from personal experience<br>**02** Perceived benefits of ANC on pregnancy outcome from experience of other women<br>**03** Husband/family decision<br>**04** Based on information by SAHIYA<br>**05** Assurance from community health worker/SAHIYA about good care at facility<br>**06** Awareness of cash benefit for registration for ANC/MJSSA-JSY scheme<br>**07** Because of a health condition<br>**08** Other (specify) ________		

	Primary reason			
	Secondary reason			
49	Why did you not attend ANC for your pregnancy?<br><br>First ask her un-promptly and based on her response note the primary and secondary reason.<br>If she is unable to answer, prompt the response.	01 No knowledge of health benefit of ANC<br>02 Place was too far and no transport<br>03 No one to accompany me<br>04 No one to look after other children at home<br>05 Too expensive to attend<br>06 Presence of male staff at facility<br>07 Fear of abusive behaviour / scolding by provider<br>08 No privacy<br>09 Other: (specify)_______		
	Primary reason			
	Secondary reason			

#	Question	Coding Categories	Skip to	Answers
**INTRAPARTUM CARE (Check 15)**				
**Intentional Place of delivery** *[For both Home and Facility delivery]*				
50	Place of delivery	**Intention** / **Actual**<br>01 Facility / Facility<br>02 Facility / Home<br>03 Home / Facility<br>04 Home / Home	 -51<br>-51, 52<br>-53, 54<br>-53	
If woman initially intended **Facility** delivery				
51	What were your reasons for wanting to deliver in a facility?<br><br>First ask her un-promptly and based on her response note the primary and secondary reason.<br>If she is unable to answer, prompt the response.	01 Perception of better pregnancy outcome with institutional delivery<br>02 Quick referral in case of emergency<br>03 Presence of doctors and nurses at the facilities<br>04 Adequate resources (medicine)<br>05 Husband/family decision<br>06 Assurance from community health worker/SAHIYA about good care at facility<br>07 Awareness of cash benefit institutional delivery[MJSSA-JSY scheme]<br>08 Centre near home<br>09 Lack of space at home<br>10 Because of a health condition<br>11 Other ___________		
	Primary reason			
	Secondary reason			
If original intention was **Facility** delivery but woman ended up delivering at **Home**				
52	How did you end up delivering at home?	01 Unexpected delivery so there was no time		

	First ask her un-promptly and based on her response note the primary and secondary reason.<br>If she is unable to answer, prompt the response.	02 There was no available transport<br>03 Absence of male /family members who could have helped arrange for transport<br>04 Was too weak and so could not go to facility to deliver<br>05 Other: ________		
	Primary reason			
	Secondary reason			
If woman initially intended **Home** delivery				
53	Why did you prefer home delivery?<br>First ask her un-promptly and based on her response note the primary and secondary reason.<br>If she is unable to answer, prompt the response.	01 No perceived health benefit of institutional delivery over home delivery<br>02 Dai lives close enough to my home<br>03 Husband/family decision<br>04 Too expensive to access<br>05 Presence of male staff at facility<br>06 Fear of abusive behaviour / scolding by provider<br>07 Fear of clinical interventions like injections and instrumentation<br>08 Unsure about availability of transport at night<br>09 Better comfort and privacy with home delivery<br>10 No one to look after other children at home<br>11 Other: _______		
	Primary reason			
	Secondary reason			
If original intention was **Home** delivery but woman ended up delivering at a **Facility**				
54	Why did you end up delivering at the facility?<br>First ask her un-promptly and based on her response note the primary and secondary reason.<br>If she is unable to answer, prompt the response.	01 Dai was unavailable<br>02 Developed a complication<br>03 Husband/family decision<br>04 Assurance from community health worker/SAHIYA about good care at facility<br>05 Lack of space at home<br>06 Other ___________		
	Primary reason			
	Secondary reason			
55	Who primarily conducted your delivery?	01 Doctor<br>02 Nurse<br>03 ANM<br>04 Dai<br>05 Family member<br>06 Friend/Neighbour<br>07 Other (Specify)		

		**Accessibility of health facility**<br>*(Facility Delivery Only)*		
56	What type/level of facility was this?	01 Government- sub-centre<br>02 PHC<br>03 CHC<br>04 District hospital		
57	How did you get to this facility?	01 On foot<br>02 Vehicle (paid)<br>03 Vehicle (free)		
58	How long did it take you to reach this facility?	01 More than 2hrs<br>02 1hr to 2hrs<br>03 30mins to1hr<br>04 Less than 30mins		
59	Is the facility easily accessible at night (or in emergency)?	01 Yes<br>02 No		
		**Structure**<br>*[Facility Delivery only]*		
60	Did the structure appear well maintained?	01 Yes<br>02 No		
61	Were toilets clean for patient use?	01 Yes<br>02 No		
62	Was there a clean delivery table in the labour/delivery room?	01 Yes<br>02 No		
		**Supplies**<br>*[Both Home and Facility Delivery]*		
63	How much did you have to pay?			
	i. Transportation			
	ii. Drugs and Injections			
	iii. Tests			
	v. Payment to Staff			
	vi. Other			
		**Human Resources**<br>*[Facility delivery only]*		
64	On your arrival was there a doctor at the facility?	01 Yes<br>02 No		
65	On your arrival Was there a nurse/ ANM at the facility?	01 Yes<br>02 No		
		**Emotional Support**<br>*[Facility delivery: only]*		
66	Who accompanied you to the facility?	01 SAHIYA<br>02 family/friend<br>03 Husband<br>04 None		
67	Did your SAHIYA assist you with your registration?	01 Yes<br>02 No		
68	Were you made to feel comfortable by all the staff/Dai/attendant at the facility/attending your delivery?	01 Yes<br>02 No		
69	Who was with you in the delivery room?	01 SAHIYA<br>02 Family / friend<br>03 None<br>04 Both (Sahiya, Family)		
70	What was the gender of the person who did your delivery?	01 Male<br>02 Female		
71	Would you have been more comfortable if the staff was/were a different gender?	01 Yes<br>02 No		
		**Cognitive Support**<br>*[Both Home and Facility delivery]*		
72	Did your birth attendant communicate with you about:			
	i. Progress of your labour	01 Yes		

		02 No		
	ii. Procedures he/she needed to do	01 Yes<br>02 No		
	iii. Pain management	01 Yes<br>02 No		
73	Do you think the entire information was adequate to your needs?	01 Yes<br>02 No		
74	Were/was the staff/Dai/birth attendant willing to answer your questions?	01 Yes<br>02 No		
**Promptness**<br>*[Both Home and Facility delivery]*				
75	How long did you have to wait before you were attended to by the person who delivered you?	01 More than 1hr<br>02 30mins to 1hr<br>03 Less than 30mins		
76	Was there any emergency/complication with the labour and delivery	01 Yes<br>02 No		
77	Were you Satisfied with the time taken to respond to your emergency?	01 Yes<br>02 No		
78	**Pain Management**<br>How was your pain managed?	01 Oral medications<br>02 Injections<br>03 Massage<br>04 Nothing was done<br>05 Other (specify) ________		
**Confidentiality and Privacy**<br>*[Both Home and Facility delivery]*				
79	Were you satisfied with the amount of privacy you were offered?	01 Yes<br>02 No		
**Inter-personal aspect of care**<br>*[Both Home and Facility delivery]*				
80	Did any staff/SAHIYA/Dai/attendant ever speak to you in a way that upset you? (e.g. shouting, using abusive language etc)	01 Yes<br>02 No		Could probe
81	Did you feel comfortable/free to discuss all your concerns with your attendant?	01 Yes<br>02 No	If No, ask 82	
82	If NO, What do you think would have made you more comfortable with your attendant?	01 female gender<br>02 male gender<br>03 someone I am familiar with<br>04 someone more friendly<br>05 other ________		probe
83	Do you think the staff/Dai/birth attendant spent enough time with you during your labour	01 Yes<br>02 No		
**Outcome**<br>*[Both Home and Facility delivery]*				
84	i. How would you rate your health immediately after childbirth?<br><br>ii. If poor and very poor health of yours, why?	01 Very good<br>02 Good<br>03 Average<br>04 Poor<br>05 Very poor		
85	i. How would you rate your baby's health immediately after	01 Very good<br>02 Good		

	childbirth?<br><br>ii. If poor and very poor health of your newborn, why?	03 Average<br>04 Poor<br>05 Very poor		

POSTNATAL CARE (Check 16)<br>**User**: Those received service from health system provider (doctor, nurse, ANM, ASHA)<br>**Non user** : received service from Dai, family members				
**#**	**Question**	**Coding Categories**	**Skip to**	**Answers**
		**Background Information**<br>*[All participants]*		
86	Did anyone check on you and your baby's health within 24 hrs after delivery?	01 Yes<br>02 No	If No, 89	
87	Where was this first check up done within 24 hour?	01 Facility<br>02 Home		
88	Who often checked on you during first 24 hour?	01 SAHIYA<br>02 Dai<br>03 Health professional (Doctor, Nurse, ANM)		
		**Emotional Support**<br>*[Both PNC Users and Non-Users]*		
89	Who primarily encouraged you with taking care of yourself and your baby during the postpartum period?	01 Husband<br>02 Other family member(s)<br>03 Friend(s)/Neighbour(s)<br>04 SAHIYA<br>05 Dai<br>06 Health professional (Doctor, Nurse, ANM)		
		**Cognitive Support**<br>*[PNC Users only]*		
90	During your check ups, did the staff/SAHIYA/Birth Attendant ever discuss with you on (who is your primary care provider):			
	i. The feeding of your baby?	01 Yes<br>02 No		
	ii. Your own nutrition?	01 Yes<br>02 No		
	iii. Child spacing (family planning)	01 Yes<br>02 No		
	iv. Your hygiene?	01 Yes<br>02 No		
	v. Your baby's hygiene? [bathing the baby, cord care]	01 Yes<br>02 No		
	vi. Immunisation of your baby?	01 Yes<br>02 No		
91	Do you think the entire information was adequate to your needs?	01 Yes<br>02 No		
92	Were/was the staff/SAHIYA/Birth Attendant interested and willing to answer your questions?	01 Yes<br>02 No		
		**Promptness**<br>*[Both PNC Users and Non-Users]*		
93	Was there any emergency/complication noticed in the postpartum period?	01 Yes<br>02 No	If No, 95	
94	If yes, Were you happy with the time taken to respond to your	01 Yes<br>02 No		

	emergency?			
**Inter-personal aspect of care** *[PNC Users Only]*				
95	Did any staff/SAHIYA/Birth Attendant ever speak to you in a way that upset you during post partum period?	01 Yes<br>02 No		
96	Did you feel comfortable/free to discuss all your concerns with your SAHIYA /other staff?	01 Yes<br>02 No	-98<br>-97	
97	If no, why or what do you think would have made you more comfortable with your attendant?	01 female gender<br>02 male gender<br>03 someone I am familiar with<br>04 someone more friendly<br>05 other _______		
98	How many PN visits were made within 6 weeks after delivery? Specify number			
99	Do you think your contact time with the staff/SAHIYA on each visit was:	01 too much<br>02 too little<br>03 just enough		
100	Do you think the number of visits was:	01 too much<br>02 too little<br>03 just enough		
**Outcome** *[Both PNC Users and Non-Users]*				
101	i. How would you rate your health after the PN period?<br><br>ii. If poor and very poor health of yours, why?	01 Very good<br>02 Good<br>03 Average<br>04 Poor<br>05 Very poor		
102	i. How would you rate your baby's health after the PN period?<br><br>ii..If poor and very poor health of your new born, why?	01 Very good<br>02 Good<br>03 Average<br>04 Poor<br>05 Very poor		
**JSY SYSTEM** *[Both PNC Users and Non-Users]*				
103	Are you aware of Rs 1400 given to deliver at health facility?	01 Yes<br>02 No		
104	When did you become aware of the JSY system?	01 During this pregnancy<br>02 During previous pregnancy<br>03 Other ____________		
105	From where you become aware of it?<br>Primarily from whom	01 SAHIYA<br>02 Hospital staff<br>03 Friend/Neighbour<br>04 Family member<br>05 Radio/TV<br>06 Other ______________		
106	Have you received the Rs 1400?	01 Yes<br>02 No	-107<br>-109	
107	If yes, how much did you receive?			
108	When did you receive it?	01 On day of delivery<br>02 Following day after delivery<br>03 Within one week of delivery<br>04 Within one month of delivery<br>05 Later than one month after delivery		
109	If no to 106 , were you told	01 Yes		

	anything about Why you were not given:	02 no		
**For women who intended Facility delivery**				
110	Will you still be willing to give birth in a facility if there is no Rs 1400?	01 Yes<br>02 No		
111	Will you still be willing to give birth in a facility if the JSY money is reduced to Rs 700	01 Yes<br>02 No		
**For women who intended to have Home delivery**				
112	Will you still be willing to give birth at home if you are promised Rs 1400?	01 Yes<br>02 No		
113	Will you still be willing to give birth at home if JSY money is increased to Rs 2800(2times)?	01 Yes<br>02 No		
**Experience based on previous place of Delivery (only for multigravida,** include live birth, still births, miscarriage)				
114	How do you compare this experience with the last pregnancy before this?	01 This one was much better<br>02 this one was slightly better<br>03 they were the same<br>04 this one was worse<br>05 this one was much worse		
**Decision about place of delivery for next child**<br>(if she doesn't intend to have next child, ask what she would recommend to a friend)				
115	Will you like to delivery in health facility again?	01 Yes<br>02 No	-116<br>-117	
116	Which of these is the reason you will like to give birth in the facility again?<br>First ask her un-promptly and based on her response note the primary and secondary reason.<br>If she is unable to answer, prompt the response.	01 Good accessibility of the facility<br>02 Good supplies<br>03 Good structure<br>04 Good care (cognitive, emotional support, inter-personal care, skill level)from providers<br>05 reasonable cost<br>06 perceived health benefits for me and my baby<br>07 Adequate staff<br>08 More comfort<br>09 More privacy<br>10 Other ___	If yes for Q.115	
	Primary Reason			
	Secondary Reason			
117	Why will you not want to give birth in the facility ?<br><br>First ask her un-promptly and based on her response note the primary and secondary reason.<br>If she is unable to answer, prompt the response.	01 Poor accessibility of the facility<br>02 poor supplies<br>03 poor structure<br>04 poor care (cognitive, emotional support, inter-personal care, skill level)from providers<br>05 too expensive<br>06 No perceived health benefits for me and my baby<br>07 fewer staff<br>08 too many male staff<br>09 No comfort<br>10 No privacy<br>11 Other ___________	If No for Q115	
	Primary Reason			
	Secondary Reason			

Maternal satisfaction with delivery care scale

Q.1 : Whether or not you were satisfied with the care received (item)
Q 2 : Then were asked about their level of satisfaction or dissatisfaction.

Encircle the appropriate response
1. Fully satisfied
2. Somewhat satisfied
3. Neither satisfied nor dissatisfied
4. Somewhat dissatisfied
5. Fully dissatisfied

1.	Antenatal preparation for this delivery by the care provider (e.g. ANM/ Nurse/ doctor/ Dai)	1	2	3	4	5
2.	Waiting time since the care provider (e.g. ANM/ Nurse/ doctor/ Dai) arrival at the place of delivery after beginning of contractions	1	2	3	4	5
3.	Respect and dignity shown by the care provider during delivery (e.g. ANM/ Nurse/ doctor/ Dai)	1	2	3	4	5
4.	Support provided by the family during delivery	1	2	3	4	5
5.	Care of the provider (e.g. ANM/ Nurse/ doctor/ Dai) during delivery	1	2	3	4	5
6.	Pain relief during delivery	1	2	3	4	5
7.	Time that the care provider (e.g. ANM/ Nurse/ doctor/ Dai) devote to their patients during delivery	1	2	3	4	5
8.	Health advices given by the care provider (e.g. ANM/ Nurse/ doctor/ Dai) to look after the newborn baby	1	2	3	4	5
9.	Opportunity given to you to clarify doubts about the care of the newborn	1	2	3	4	5
10.	Competency of the care provider (e.g. ANM/ Nurse/ doctor/ Dai) in providing care to both you and your baby	1	2	3	4	5
11.	Health condition of your newborn baby	1	2	3	4	5
12.	Health condition of yourself after delivery	1	2	3	4	5
13.	The care you received during the Antenatal period	1	2	3	4	5
14.	The care you received during the delivery	1	2	3	4	5
15.	The care you received during the Postnatal period	1	2	3	4	5
